# Supplementary material for: MetaRibo-Seq measures translation in microbiomes
Source: Nat Commun. 2020 Jun 29;11:3268. doi: 10.1038/s41467-020-17081-z (PMC7324362; doi:10.1038/s41467-020-17081-z)
Supplement: Supplementary file 10 — Supplementary Data 7 [file 41467_2020_17081_MOESM10_ESM.zip › File2/Confidence_High_Taxonomy/183164_out.krona.html]

Javascript must be enabled to view this page.

members
magnitude
magnitudeUnassigned
count
unassigned
taxon
rank

183164\_out

2

2
2
superkingdom

2
976
phylum

200643
class
2

171549
order
2

815
family
1

genus
816
1

SRS142890\_contig\_number\_contig-100\_20104.156483

1
family
171552

1
838
genus

60133
species
1

SRS017076\_contig\_number\_contig-100\_19275.19275.19275
